# Functional Foods from Black Rice (*Oryza sativa* L.): An Overview of the Influence of Drying, Storage, and Processing on Bioactive Molecules and Health-Promoting Effects

**DOI:** 10.3390/foods13071088

**Published:** 2024-04-02

**Authors:** Lázaro Cañizares, Silvia Meza, Betina Peres, Larissa Rodrigues, Silvia Naiane Jappe, Paulo Carteri Coradi, Maurício de Oliveira

**Affiliations:** 1 Department of Agroindustry Science and Technology, Federal University of Pelotas, Pelotas 96010-900, Brazil; lazarocoosta@hotmail.com (L.C.); silvialrmeza@gmail.com (S.M.); betinabuenop@gmail.com (B.P.); larissaalvesralf@gmail.com (L.R.); jappesilvia@gmail.com (S.N.J.); mauricio@labgraos.com.br (M.d.O.); 2 Laboratory of Postharvest (LAPOS), Campus Cachoeira do Sul, Federal University of Santa Maria, Avenue Taufik Germano, 3013, Universitário II, Cachoeira do Sul 96503-205, Brazil

**Keywords:** industrial application, functional food, phenolic compounds, health benefits

## Abstract

Black rice *(Oryza sativa*) stands out for its high content of bioactive compounds with functional properties that play an important role in health benefits. The phytochemical level is affected by industrial processing due to its instability to the hydrothermal process. Studies about the influence of industrial processing on the phytochemical profile of black-rice-based foods are still scarce. This study carried out a comprehensive review of the influence of industrial applications on the bioactive compounds in food products based on black rice and their health-promoting effects. Most industrial processes such as drying, storage, cooking, and extrusion affect phytochemical content and antioxidant capacity. Alternatively, technologies such as fermentation, UV-C irradiation, and sprouting can maintain or improve the phytochemical content in black rice products.

## 1. Introduction

Rice consumption worldwide predominantly favors brown pericarp cultivars, albeit in their polished form. White rice is the result of processing whole rice, where the bran and germ are removed, leaving only the endosperm. This process removes many of the nutrients found in whole rice, such as fiber, vitamins, and minerals, making white rice less nutritious in comparison. On the other hand, pigmented rice such as black rice, red rice, and purple rice retains its bran and germ, giving it a characteristic color and a richer nutritional profile. These cultivars have attracted attention from researchers, industries, and consumers, primarily due to their unique subgroups of polyphenols, which hold potential health benefits [1].

The exact origin of different varieties of pigmented rice is not clearly defined, but many have their roots in Asian regions such as India, China, and Thailand. These varieties have been cultivated for centuries, and are an integral part of the cuisine and culture of these regions. The importance of pigmented rice varieties goes beyond their nutritional properties. They play a significant role in diversifying agricultural production and promoting healthier, more balanced diets. Consuming these varieties can help increase the intake of antioxidants and other essential nutrients, contributing to better overall health and well-being [2].

Black pericarp rice, in particular, is noted for its high concentrations of bioactive compounds, especially flavonoids and phenolic acids [1]. The primary flavonoid found in black rice grains is anthocyanin, a group of reddish to purple flavonoids predominantly located in the aleurone layer [3]. The anthocyanin content is closely linked to the rice pericarp color. The greater intensity of the grains is attributed to high flavonoid levels, demonstrating a positive correlation with antioxidant activity. The antioxidant capacity of black rice is associated with health-promoting functions, including the prevention of hypocholesterolemia, cardiovascular disease, diabetes, obesity, and cancer [4,5]. Among the phenolic acids, ferulic acid, p-coumaric acid, vanillic acid, gallic acid, rutin, quercetin, syringic acid, and protocatechuic acid are the main compounds found in black rice. However, the content of these phytochemicals can vary according to post-harvest and industrial processes [1,6].

Post-harvest and industrial processing play a crucial role in grain quality. They directly affect food safety, shelf life, nutritional value, sensory quality, and acceptance of the final product. Post-harvest processing, such as drying and storage, is essential for preserving grain quality, preventing deterioration, and losses. On the other hand, industrial processing, including polishing, milling, sprouting, cooking, baking, extrusion, and fermentation methods, directly influences the texture, flavor, aroma, and appearance of foods, as well as altering their nutritional composition and content of bioactive compounds. Both processes are essential to meet the quality and food safety standards required by consumers and regulators [7,8,9].

Moreover, whole black rice or its by-products can be utilized in the development of functional foods through biotechnological processes, offering high added value owing to their nutritional and phytochemical composition. The creation of active foods based on black rice represents a sustainable approach for repurposing industrial rice waste, such as rice bran [7]. The global demand for plant-based products has been rapidly increasing, driven by the necessity to provide healthier food options for a growing population. However, post-harvest and industrial processes methods used to manufacture black rice products can impact the content of bioactive compounds due to the diverse processing conditions employed. In this context, this review aims to provide a comprehensive overview of the current literature regarding the influence of drying, storage, and processing on the bioactive molecules present in black pigmented rice.

### Review Structure

This study conducted a literature review on the effects of industrial pre-processing and processing of black-rice-based foods on the bioactive compounds of black rice. The research cited in this review was gathered by searching the Web of Science database for studies published from 2017 to 2023. The selection of the database from 2017 to 2023 for this review study was based on several factors, including the need for recent and comprehensive research, the availability and breadth of literature in the Web of Science database, the balance between inclusivity and relevance in the chosen timeframe, and the importance of ensuring the comparability and consistency of the included studies.

**Figure 1:**
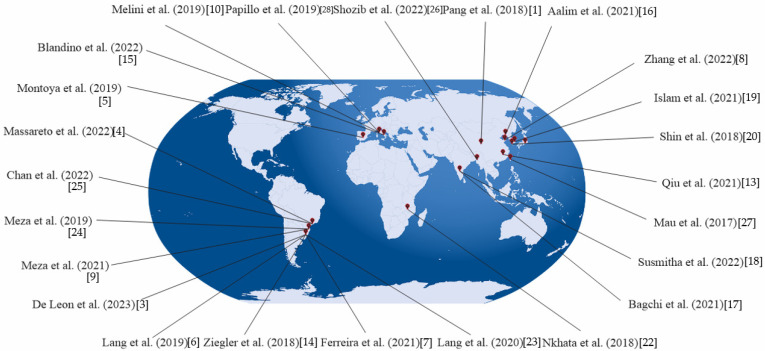
Geographical distinction of the studies about black rice presented in this review.

The search terms included “black rice composition”, “bioactive compounds in black rice”, “phytochemicals in black rice”, “industrial processing of black rice”, “black rice drying”, “black rice storage”, “black rice polishing”, “black rice milling”, “black rice sprouting”, “black rice cooking”, “black rice baking”, “black rice extrusion”, “black rice fermentation”, “development of black rice-food products”, “food products based on black rice”, and “phytochemicals in black rice-based foods”. Following a thorough and current search, 31 studies were included in this review. A geographical distinction presented in Figure 1 was made to clearly visualize the locations where black rice studies were conducted, which can aid in understanding regional variations, cultural and dietary contexts, research focuses, and the global significance of black rice research. Among them, ten studies were conducted in Brazil, four in China, three in Italy, two in Korea, one in Bangladesh, one in Taiwan, one in India, and one in France.

## 2. Black Rice Fractions as a Source of Phytochemicals

**Figure 2:**
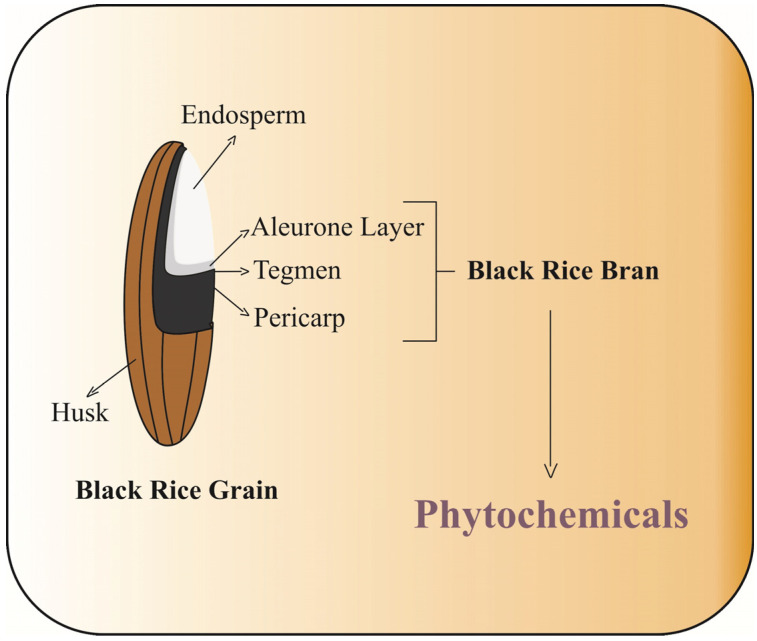
Bioactive molecules distribution in black rice grain (*Oryza sativa*).

The phytochemical compounds are mainly distributed in the outermost fraction of the rice grain, such as the aleurone, bran, and pericarp, referred to as rice bran (Figure 2). In order to gain a better understanding of the distribution of phytochemicals in black rice grains, the study conducted by Zhang et al. [8] showed the contents of phytochemical in different milling black rice fraction (BF), which collectively represent approximately 2% of the whole grain.

The BF1 fraction represents the outermost layer of the grain, while the remaining fractions (BF2, BF3, BF4, and BF5) consist of the innermost layers. Studies have shown that the concentration of phenolic compounds, flavonoids, and anthocyanins, both free and bound, is higher in the outermost layers of black rice [1,3,9]. The BF1 fraction (2% of the outermost bran) contained 4436.12 and 658.76 mg GAE·100 g^−1^ of free and bound phenolic compounds, respectively, 13,106.16 and 902.25 mg EC·100 g^−1^ of free and bound flavonoids, respectively, and 3158.45 mg CGE·100 g^−1^ of total anthocyanins.

The main phenolic acids found in whole black rice bran and BF1 were ferulic acid (7.23 and 96.97 µg g^−1^, respectively), p-coumaric acid (3.29 and 50.27 µg·g^−1^, respectively), gallic acid (5.05 and 36.43 mg·100 g^−1^, respectively), and vanillin (3.12 and 12.42 µg·g^−1^, respectively). The authors concluded that 84.8% of the phenolic compounds, 77.5% of the flavonoids and 73.9% of the anthocyanins are present in 2% of the grain (outer layer), making it an excellent source of anthocyanins and phenolic acids with effective bioactivity for several industrial applications.

When comparing these values with brown rice, an increase of 657.6% in total phenolic compounds, 1024.18% in total flavonoids, and 1206.6% in total anthocyanins was observed [10]. Zhang et al. [8] also quantified the main anthocyanins found in black rice. The levels of cyanidin-3-O-glucoside ranged from 203.18 to 2568.63 mg·100 g^−1^, peonidin-3-O-glucoside ranged from 7.94 to 95.46 mg·100 g^−1^, malvidin-3-O-glucoside ranged from 1.50 to 16.02 mg·100 g^−1^, and delphinidin-3-glucoside ranged from 0.27 to 4.18 mg·100 g^−1^.

The studies provide valuable insights into the distribution of phytochemicals in black rice grains. The outermost fraction of the grain, known as rice bran, contains a higher concentration of phenolic compounds, flavonoids, and anthocyanins compared to the inner layers. This finding underscores the nutritional significance of the outer layers of black rice. The studies also highlight the presence of key phenolic acids in black rice bran and the BF1 fraction, indicating their potential health benefits. Moreover, the comparison with brown rice demonstrates a substantial increase in bioactive compounds in black rice, further emphasizing its nutritional superiority. Overall, the research sheds light on the bioactive molecule distribution in black rice, and underscores its potential as a functional food with industrial applications.

## 3. Effect of Post-Harvest Processes on the Bioactive Molecules of Black Rice

The drying and storage processes are pivotal for maintaining the quality of grains, including black rice. These processes are essential for preserving the nutritional content of grains by inhibiting the growth of mold, bacteria, and other microorganisms that can degrade nutrients. Additionally, proper drying and storage prevent spoilage, which can lead to the formation of toxins and off-flavors, ensuring that grains remain safe for consumption. Moreover, these processes help maintain the texture and flavor of grains, as they prevent the development of undesirable textures and flavors that can occur with improper storage [11].

Furthermore, drying and storage methods play a critical role in preventing insect infestation, which can damage grains and reduce their quality. By properly drying grains, the moisture content is reduced, making them less susceptible to insect infestation. Additionally, these processes extend the shelf life of grains, allowing them to be stored for longer periods without compromising their quality. Overall, the proper drying and storage of grains, such as black rice, are essential for ensuring their safety, nutritional quality, and longevity [12]. However, post-harvest processes such as drying and storage can affect the levels of bioactive molecules in black rice mainly in the content of total phenolic, flavonoids, anthocyanins, and antioxidant capacity (Table 1). In this section, we will address the main impacts on the phytochemical profile of black rice after drying and storage.

### Drying and Storage

Black rice is harvested with high moisture content (18–25%) and therefore needs to be dried to ensure safety and extend shelf life. Lang et al. [6] analyzed the effects of drying temperature (20, 40, 60, 80, and 100 °C), storage time (12 months), and storage conditions (normal atmosphere, nitrogen atmosphere, and vacuum atmosphere) on the phenolic compounds of black rice. After drying, a decrease in total phenolic compounds was observed with increasing temperature, along with a reduction in total free flavonoids when exposed to drying temperatures exceeding 60 °C. However, during storage, an increase in the free fraction of flavonoids was observed, particularly in a normal atmosphere. This is likely due to the higher presence of oxygen in conventional storage, which may have favored grain metabolism and, consequently, the action of enzymes associated with the hydrolysis of the grains’ structural constituents, releasing the bound fraction of these compounds. Protocatechuic acid and the quercetin flavonoid were the only free phenolics that did not decrease with increasing drying temperature. In contrast, ferulic acid, p-coumaric acid, gallic acid, caffeic acid, and gallic acid all showed a reduction. Interestingly, protocatechuic acid exhibited an increase in content with rising drying temperature, possibly due to the degradation of anthocyanin cyanidin-3-O-glycoside. 

Moreover, Ziegler et al. [14] evaluated the phenolic composition of black pericarp rice during six months of storage at temperatures of 16, 24, 32, and 40 °C. There were no significant differences in the total free phenolic compounds between the initial storage period (23.1 mg GAE·g^−1^) and after 6 months at temperatures of 16, 24, 32, and 40 °C (22.8, 22.1, 23.0, and 22.8 mg GAE·g^−1^, respectively). Similar behavior was found for the total anthocyanin content in black rice, with no differences observed between the initial storage period (2178.2 mg C3GE·kg^−1^) and after six months at temperatures of 16, 24, 32, and 40 °C (2186.2, 2185.6, 2183.7, and 2186.8 mg C3GE·kg^−1^, respectively). When the individual free phenolic compounds were analyzed, p-coumaric acid was not detected at the beginning of storage or after six months at 16 °C, but levels of 1.0 µg·g^−1^ were found when stored at 40 °C. Furthermore, there was an increase in the concentration of ferulic acid from 1.7 µg·g^−1^ at the beginning of storage to 6.3 µg·g^−1^ after six months at 40 °C. This rise may be attributed to the decomplexation of these compounds under high drying temperatures.

On the other hand, new technologies can be applied to preserve the phytochemical profile in black rice during storage. Ferreira et al. [7] investigated the impact of UV-C radiation on the phenolic compounds of black rice over one and three-hour exposures, followed by a six-month storage period. The study revealed an increase in gallic acid levels from 1.0 to 1.1 µg·g^−1^, hydroxybenzoic acid from 2.5 to 3.3 µg·g^−1^, coumaric acid from 0.8 to 1.1 µg·g^−1^, and ferulic acid from 2.8 to 3.4 µg·g^−1^ in black rice grains without and after 3 h of UV-C radiation exposure, respectively. This suggests that UV-C radiation may break bonds and enhance the extractability of phenolic compounds, previously associated with the cell wall [10].

The main findings of these studies indicate that black rice, harvested with high moisture content, requires drying to ensure safety and extend shelf life. Analysis of the effects of drying temperature, storage time, and storage conditions on the phenolic properties of black rice showed a reduction in total phenolic compounds and free flavonoids with increasing drying temperature, while an increase in the free fraction of flavonoids was observed during storage, especially in a normal atmosphere. This increase may be attributed to the higher presence of oxygen, which favors the metabolism and action of enzymes associated with the release of phenolic compounds. Additionally, studies showed that some free phenolics did not decrease with increasing temperature, while others showed reduction. New technologies, such as UV-C radiation, can also be applied to preserve the phytochemical profile in black rice during storage, showing significant increases in some phenolic acids after exposure to UV-C radiation. These results highlight the importance of drying and proper storage to maintain the quality and nutritional benefits of black rice.

## 4. Effect of Industrial Processing on the Bioactive Molecules of Black Rice

The industrial processing of black rice can significantly impact its bioactive molecules. Processing methods such as milling, polishing, and cooking can alter the content and availability of bioactive compounds in black rice. For example, milling and polishing remove the outer layers of the grain, which are rich in phenolic compounds, flavonoids, and anthocyanins, reducing the overall content of these beneficial compounds in the final product. Cooking can also affect the bioactive compounds, as some are sensitive to heat and may degrade during the cooking process. However, certain processing methods, such as fermentation, can enhance the bioavailability of these compounds by breaking down complex molecules into simpler forms that are more easily absorbed by the body. Overall, the industrial processing of black rice can have both positive and negative effects on its bioactive molecules, highlighting the importance of choosing processing methods that preserve or enhance the nutritional value of this valuable grain. 

Black pigmented rice can be used as ingredient in diverse food products such as cakes, breakfast cereals, snacks, pasta, beverages, and breads. However, the industrial processing used to produce black rice-based foods can affect the content of bioactive molecules mainly the content of total phenolic, flavonoids, anthocyanins, and antioxidant capacity (Table 1). In this section, we will discuss the main industrial processes to which black rice is subjected, such as polishing, popping, puffing, beating, boiling, sprouting, cooking, baking, extrusion, and fermentation, and how these processes affect the phytochemical quality of black rice.

### 4.1. Polishing, Popping, Puffing, Beating, and Boiling

The processes of polishing, popping, puffing, beating, and boiling are essential for food processing, especially grains and cereals. Polishing, for example, is used to remove the outer husk or bran of grains like rice, resulting in a more refined and white product. On the other hand, popping and puffing involve the use of heat and pressure to turn grains into light and crispy products, such as popcorn and “puff” cereals. Beating is a technique used to incorporate air into ingredients like eggs or cake batter, creating a lighter and softer texture. Boiling, meanwhile, is a cooking method in boiling water that softens foods and alters their texture and flavor, being essential in various dishes and culinary preparations.

Bagchi et al. [17] investigated the impact of various processing methods, including raw polished rice, popped rice, puffed rice, beaten rice, and cooked rice, on the flavonoid content of four black pigmented rice cultivars (Kalobhat, Chakha, Mamihunger, and Manipuri Black) grown in Odisha, India (Table 1). The individual free phenolics in raw polished rice, cooked for 18–20 min, included ferulic acid (0.0–248.8 mg 100 g^−1^), gallic acid (454.5–688.0 mg·100 g^−1^), kaempferol (50.7–99.3 mg·100 g^−1^), myricetin (30.0–100.8 mg·100 g^−1^), PAA (0.0–118.8 mg·100 g^−1^), and vanillic acid (44.5–81.5 mg·100 g^−1^). The total anthocyanin content was 0.8–3.9 mg·100 g^−1^, and the antioxidant activity was 0.08–0.16 mM AAE·100 g^−1^ using an ABTS assay. When analyzing the individual free phenolics in beaten rice, which was exposed to 60 °C for 45 min, the following concentrations were found: ferulic acid (1224.0–1789.2 mg·100 g^−1^), kaempferol (56.3–192.4 mg·100 g^−1^), myricetin (116.2–310.0 mg·100 g^−1^), gallic acid (1099.1–1560.5 mg·100 g^−1^), PAA (0.0–855.0 mg·100 g^−1^), and vanillic acid (12.4–379.1 mg·100 g^−1^). Total anthocyanins were measured at 16.72–33.09 mg·100 g^−1^, and antioxidant activity was recorded at 0.27–0.32 mM AAE·100 g^−1^ (ABTS assay).

In the analysis of individual free phenolics in popped rice, which was exposed to temperatures exceeding 177 °C for 40–50 s, the following concentrations were observed: gallic acid (1592.5–2782.3 mg·100 g^−1^), ferulic acid (157.4–1635.5 mg·100 g^−1^), kaempferol (29.3–109.5 mg·100 g^−1^), PAA (0.0–984.8 mg 100 g^−1^), vanillic acid (0.0–314.8 mg·100 g^−1^), and myricetin (91.3–799.7 mg·100 g^−1^). Total anthocyanin content ranged from 4.30 to 31.93 mg 100 g^−1^, and antioxidant activity was measured at 0.29–0.32 mM AAE·100 g^−1^ (ABTS assay). Upon analyzing the individual free phenolics in puffed rice, which underwent roasting with a 10–12% brine solution for 1–2 min at 220 °C, the following concentrations were found: gallic acid (1009.2–1517.3 mg·100 g^−1^), vanillic acid (19.2–319.7 mg·100 g^−1^), ferulic acid (383.7–1272.8 mg·100 g^−1^), kaempferol (39.6–265.6 mg·100 g^−1^), PAA (0.0–965.7 mg·100 g^−1^), and myricetin (60.9–435.9 mg·100 g^−1^). Total anthocyanins ranged from 0.44 to 17.62 mg·100 g^−1^, and antioxidant activity was measured at 0.14–0.26 mM AAE·100 g^−1^ (ABTS assay).

Upon processing, there was a reduction in apigenin, catechin, miricetin, and total flavonoids and an increase in kaempferol and luteolin. This reduction was greater in the order of polished rice, popped rice, puffed rice, beaten rice, and cooked rice, given the severity of the processes to which the rice grains were subjected. The addition of some free flavonoids (kaempferol and luteolin) occurs due to their decomplexation when subjected to processing, given their greater stability compared to other compounds.

It was possible to explore the impact of different processing methods on the flavonoid content of four black pigmented rice cultivars. They examined raw polished rice, cooked for 18–20 min, and found varying concentrations of phenolic compounds such as ferulic acid, gallic acid, kaempferol, myricetin, PAA, and vanillic acid, along with total anthocyanins and antioxidant activity. Beaten rice, exposed to 60 °C for 45 min, showed increased concentrations of these compounds. Popped rice, exposed to high temperatures, exhibited different phenolic levels, as did puffed rice, roasted with a brine solution. Processing led to a reduction in some flavonoids and an increase in others, influenced by the severity of processing methods, with the addition of certain flavonoids occurring due to decomplexation during processing, reflecting their greater stability compared to other compounds.

### 4.2. Sprouting

Sprouting refers to the process by which seeds germinate and begin to sprout, forming small seedlings. This process involves the activation of enzymes within the seed, which begin to break down the seed’s starch and protein reserves into smaller components, such as simple sugars and amino acids, which are necessary for the plant’s initial growth. During germination, significant biochemical changes also occur, including an increase in certain nutrients and bioactive compounds, such as vitamins, antioxidants, and phenolic acids, which may have health benefits when sprouted foods are consumed.

The black rice sprouting at 25 °C and 95% moisture for 3 days resulted in significant changes in total phenolic compounds, total flavonoids, anthocyanins, and antioxidant activity (Table 1). Total phenolic compounds ranged from 77.0 to 149.0 mg·100 g^−1^, while total flavonoids ranged from 36.0 to 45.0 mg·100 g^−1^. Anthocyanins showed a wide variation from 890.7 to 4582.5 mg·100 g^−1^. The antioxidant activity, evaluated using the DPPH assay, ranged from 0.06 to 0.045 mM TE·100 g^−1^. These results highlight the potential of black rice sprouting as a process capable of increasing the concentration of bioactive compounds and the antioxidant activity of the grain.

The variation in the content of bioactive molecules after germination, including flavonoids and GABA, can occur due to the activation of endogenous enzymes related to biochemical and biofunctional compounds [16]. After germination, the level of GABA in pigmented rice can increase by up to 11-fold when compared to non-germinated rice. The increase in GABA content in rice can begin during the imbibition or soaking of the grains once respiration accelerates. This process stimulates the metabolism of amino acids, leading to the production of the glutamate decarboxylase enzyme, which is activated upon water absorption by the rice grain, transforming glutamic acid into GABA. GABA plays an important role in specific reactions in the body, such as reducing stress, improving sleep, preventing brain damage, and relieving anxiety, due to its antimicrobial, anticonvulsant, and antioxidant properties [21].

In addition, the content of phenolic compounds in pigmented rice can also increase after germination due to their release caused by the action of amylolytic and proteolytic enzymes. On the other hand, pigmented rice after germination generally has a lower anthocyanin content than non-germinated rice. The reduction in anthocyanin levels can be related to the loss of pigments during the soaking process. However, alterations in the content of bioactive molecules can also depend on the crops, cultivar, sample preparation, and processing [22].

The study examined the effects of soaking and sprouting on black rice bioactive compounds. After sprouting, there was an increase in total flavonoids and a reduction in total anthocyanins in both cultivars studied. Additionally, antioxidant capacity varied between cultivars after sprouting. The variation in bioactive compounds after germination, including flavonoids and GABA, is attributed to the activation of endogenous enzymes. Germination can lead to a significant increase in GABA content, with potential health benefits such as stress reduction and improved sleep. The amount of phenolic compounds in pigmented rice may also increase after germination, while anthocyanin levels generally decrease. These changes in bioactive compounds may vary depending on the crop, cultivar, sample preparation, and processing methods.

### 4.3. Cooking

The process of cooking refers to the act of preparing food using heat, whether by boiling, frying, baking, or other methods. During cooking, foods are exposed to high temperatures, which causes physical and chemical changes in them. These changes may include the breakdown of cellular tissues, the caramelization of sugars, the denaturation of proteins, and the gelatinization of starches. The cooking process not only makes food safer to eat by killing pathogenic microorganisms, but can also enhance the flavor, texture, and digestibility of foods.

Melini et al. [10] analyzed the levels of total phenolic compounds and anthocyanins in four black rice genotypes (OTL, VNR, NRN, and ATM) grown in Italy, both before and after cooking. After cooking, there was a reduction in the levels of free phenolic compounds in all genotypes. The ATM genotype showed the highest levels of free phenolic compounds post-cooking (1384.8 mg·100 g^−1^), followed by the NRN genotype (803.7 mg·100 g^−1^), VNR genotype (624.4 mg·100 g^−1^), and OTL genotype (528.4 mg·100 g^−1^).

Also, it analyzed the content of anthocyanin cyanidin-3-O-glucoside and peonidin-3-O-glucoside in the OTL, NRN, ATM, and VNR genotypes after cooking. A reduction in cyanidin-3-O-glucoside from 7150.0 to 6550.0 mg·100 g^−1^ in the OTL genotype, from 1305.0 to 6310.0 mg 100 g^−1^ in the NRN genotype, and from 19,860.0 to 10,060.0 mg·100 g^−1^ in the ATM genotypes, whereas an increase from 8170.0 to 9350.0 mg·100 g^−1^ in VNR genotype was observed after cooking. For the anthocyanin peonidin-3-O –glucoside, a reduction was observed in all analyzed cultivars. The authors linked the great variability in the content of phenolic compounds among black rice samples to the genetic characteristics, production climate and geographic location, harvest, and post-harvest handling and/or storage [10].

In addition, Aalim et al. [16], analyzed the effect of processing (raw rice, cooked rice, roasted, roasted, and oil frying) on the phenolics of black rice (Liaoning, China). Roasting kept phenolic compounds, flavonoids, and proanthocyanins stable. Anthocyanins were reduced in all processing methods, which the smallest reduction was observed in roasted rice. Regarding the flavonoid profile, a reduction in cyanidin-3-glucoside from 156.76 to 116.18, 135.15, 36.72, and 21.64 mg·100 g^−1^, and rutin from 4.32 to 3.97, 0.75, 4.05, and 2.09 mg·100 g^−1^ was observed, and an increase in kaempferol from 1.23 to 2.87, 2.57, 1.73, and 2.28 mg 100 g^−1^, and isorhamnetin from 2.51 to 3.56, 3.01, 5.58, and 4.52 mg·100 g^−1^ was observed when we analyzed raw rice, cooked, roasted, roasted, and oil frying rice, respectively. The most abundant phenolic compound found was cyanidin-3-glucoside, which is unstable in thermal processing. Cyanidin-3-glucoside decomposes into protocatechuic acid, which can be observed by the increase in this content from 143.23 to 359.88, 247.85, 219.30, 199.42 mg·100 g^−1^, when we analyzed raw rice, cooked, roasted, roasted, and oil frying, respectively. Furthermore, an increase in lipase inhibitory activity was observed with cooking rice, and a decrease in α-amylase and lipase inhibitory activity in roasted and fried rice. In general, black rice showed greater antiglycation activity and processing further increased activity, supporting the view that consumption of black rice can regulate postprandial glucose, since cyanidin-3-glucoside is an important antihyperglycemic agent [23]. These findings underscore the favorable impact of black rice processing on nutritional management in consumers with metabolic disorders. 

Black rice, when processed, shows changes in phenolic compounds and anthocyanins, impacting its nutritional value. Cooking reduces free phenolic compounds, while some anthocyanins decrease and others increase. Variability in phenolic content is linked to genetic factors, production conditions, and handling. Different processing methods affect phenolic content differently, with roasting maintaining stability but reducing anthocyanins. The most abundant phenolic compound, cyanidin-3-glucoside, decomposes into protocatechuic acid during processing. Overall, black rice exhibits potential for managing metabolic disorders due to its antiglycation activity and the presence of antihyperglycemic agents like cyanidin-3-glucoside.

### 4.4. Baking

The process of baking refers to cooking food in an oven, usually using circulating hot air. During baking, heat penetrates the food, cooking it evenly and resulting in a distinctive texture and flavor. Baking is commonly used for baking bread, cakes, cookies, and other pastry products, as well as for roasting meats, vegetables, and other foods. The baking process may involve the use of ingredients such as flour, sugar, eggs, and yeast.

Black rice grains can be utilized in cake production [17]. The authors noted an increase in hydroxybenzoic acid (205.2%), caffeic acid (99.5%), caftaric acid (318.1%), protocatechuic acid (26.6%), and quercetin (0.8%) in the cakes compared to whole flour. This increase resulted from thermal hydrolysis during the cake production process, enhancing the extractability of these compounds, as phenolic acids are primarily found in rice’s bound fraction, linked to lignin by ether bonds through its hydroxyl groups or attached to proteins and structural carbohydrates through their carboxylic group [17]. However, the processing instability varied among the compounds we analyzed, with a reduction of 21.14% in p-coumaric acid and 23.53% in ferulic acid observed, due to their high susceptibility to thermal degradation [6].

The use of pigmented rice to replace conventional rice in typical recipes involved using five different cultivars of black rice (Chakhao, Kalobhat, Mamihunger, Manipuri Black, and Poireiton) as a substitute for white rice in the formulation of idli, a typical Indian cake. Cakes formulated with black rice showed higher contents of phenolic acids (12-fold), flavonoids (88-fold), and antioxidant activity (44-fold) compared to traditional cakes based on white rice [24].

Black rice grains are suitable for cake production. When compared to whole flour, cakes made with black rice showed increased levels of hydroxybenzoic acid, caffeic acid, caftaric acid, protocatechuic acid, and quercetin. This increase is attributed to thermal hydrolysis during cake production, enhancing the extractability of these compounds. However, the processing instability varied among compounds, with p-coumaric acid and ferulic acid showing reductions due to their high susceptibility to thermal degradation. Additionally, using black rice cultivars as a substitute for white rice in idli formulation led to cakes with significantly higher levels of phenolic acids, flavonoids, and antioxidant activity compared to traditional white rice cakes.

### 4.5. Extrusion

Another technology widely used in the development of new products derived from black rice is extrusion processing. Extrusion is a processing technique used in the food industry to produce food in a dough-like form, such as breakfast cereals, snacks, pasta, and pet foods. It involves forcing a material through an extrusion die using heat, pressure, and mechanical movement. During extrusion, the material is subjected to high temperatures and pressures, resulting in physicochemical changes that alter the product’s structure. The process is capable of modifying food properties such as texture, flavor, digestibility, and nutritional density, and allows for the addition of functional ingredients. Extrusion is also effective in reducing pathogenic microorganisms and denaturing antinutrients, improving food safety, and quality.

Blandino et al. [15] produced extruded snacks based on black rice flour. The authors observed a reduction in soluble phenolic acids from 12.8 to 8.2 mg 100 g^−1^, total anthocyanins from 181.6 to 17.3 mg C3GE·100 g^−1^, but the antioxidant capacity was not affected (2.22 and 2.20 mM TE·100 g^−1^) when compared to flour and extruded snacks, respectively. When analyzing the individual phenolic acids, a reduction in protocatechuic from 62.0 to 14.0 mg·100 g^−1^, hydroxy-benzoic from 130.0 to 56.0 mg·100 g^−1^, ferulic from 358.0 to 199.0 mg·100 g^−1^ and sinapic acid from 453.0 to 156.0 mg·100 g^−1^, while an increase in vanillic from 190.0 to 238.0 mg·100 g^−1^, caffeic from 0.0 to 150.0 mg·100 g^−1^, syringic from 170.0 to 20.0 mg·100 g^−1^, and p-coumaric from 74.0 to 129.0 mg·100 g^−1^ was observed.

Chan et al. [25] produced extruded gluten-free pasta from black rice by-products. The authors observed a reduction in total anthocyanins from 41.5 to 12.4 mg C3G·100 g^−1^ after extrusion of black rice pasta, respectively. Moreover, Meza et al. [9] produced breakfast cereals from black rice flour by extrusion technology with acceptable technological and sensory proprieties. Meza et al. [9] evaluated the impact of extrusion processing on the bioactive molecules of black pigmented rice. The authors observed a reduction in total phenolic compounds from 569.29 to 180.71 mg FAE·100 g^−1^, total flavonoids from 496.89 to 153.71 mg CE·100 g^−1^, anthocyanins from 559.41 to 105.68 mg C3GE·100 g^−1^, and antioxidant capacity from 2.85 to 0.87 mM TE·100 g^−1^ after extrusion process of breakfast cereals. The reductions occurred due to the instability of the bioactive compounds when exposed to high temperatures and pressure conditions of extrusion processing.

Qiu et al. [13] studied the effect of drum drying and extrusion in black pericarp rice on the contents of total phenolics, flavonoids, anthocyanins, and antioxidant capacity. Cyanidin-3-glucoside and peonidin-3-glucoside were the main anthocyanins found in the raw black rice sample (2745.0 and 322.0 mg·100 g^−1^, respectively). However, there was a reduction in these compounds by 35.52 and 34.16% when subjected to drum drying, and by 72.86 and 59.01% when subjected to extrusion, respectively. Thus, there was an increase in protocatechuic acid and cyanidin, since they are a degradation products of cyanidin-3-glucoside. When analyzing the total phenolic compounds, total flavonoids, and total anthocyanins, a reduction was observed from 13,882.0 to 4597.0 and 12,179.0 mg GAE·100 g^−1^ DW, from 5974.0 to 1030.0 and 1962.0 mg GAE·100 g^−1^, and from 113.75 to 68.27 and 33.68 mg C3GE·100 g^−1^ when analyzing raw, drum-dried, and extruded rice. Furthermore, the authors observed a reduction in antioxidant capacity by DPPH radical scavenging activity when black rice was exposed to drum drying (5.0 mM TE·100 g^−1^) and extrusion processing (19.0 mM TE·100 g^−1^), compared to raw black rice (29.0 mM TE·100 g^−1^). The ABTS radical scavenging activity reduced from 327.0 to 30.0 and 144.0 mM TE·100 g^−1^ when used drum drying and extrusion, respectively.

The studies on black rice processing techniques have provided valuable insights into the changes in bioactive compounds and antioxidant capacity. Blandino et al. [15] showed that while some phenolic acids and anthocyanins were reduced in extruded snacks, the antioxidant capacity remained stable, suggesting potential health benefits of these products. Chan et al. [25] demonstrated the feasibility of producing gluten-free pasta from black rice by-products, albeit with a reduction in anthocyanins. Meza et al. [9] highlighted the impact of extrusion on breakfast cereals, noting reductions in various bioactive compounds and antioxidant capacity. Qiu et al. [13] further emphasized the importance of processing methods, showing significant reductions in anthocyanins, phenolic compounds, and antioxidant capacity in drum-dried and extruded black pericarp rice. Future research could focus on optimizing processing conditions to minimize the loss of bioactive compounds and antioxidant capacity in black rice products. Additionally, studying the bioavailability and health effects of these processed products in human trials could provide valuable insights into their potential as functional foods. Developing innovative processing techniques that preserve or enhance the nutritional profile of black rice could lead to the production of healthier and more nutritious food products.

### 4.6. Fermentation

Fermentation is a metabolic process that converts carbohydrates, such as sugars and starches, into alcohol or organic acids using microorganisms like yeast, bacteria, or fungi. This process occurs without the presence of oxygen, known as anaerobic conditions. Fermentation is used in various food and beverage production processes, such as brewing beer, making wine, fermenting bread dough to rise, and producing yogurt, cheese, and sauerkraut. Fermentation can also be used to preserve foods and enhance their flavor, texture, and nutritional value.

Shin et al. [20] studied the effect of solid-state fermentation (0, 1, 2, 3, 4, and 5 days of fermentation) with Aspergillus awamori and Aspergillus oryzae in black rice bran on phenolic acid composition and antioxidant activity. The authors observed an increase in the protocatechuic acid and vanillic acid contents in the third day of fermentation from 15,116.0 and 9000.0 mg·100 g^−1^, respectively (without fermentation), to 166,060.0 and 46,200.0 mg·100 g^−1^, respectively (fermented with A. awamori), and 157,960.0 and 44,530.0 mg·100 g^−1^, respectively (fermented with A. oryzae). In the second day of fermentation, the authors observed an increase in hidroxybenzoic acid and p-coumaric acid from 300.0 and 530.0 mg·100 g^−1^, respectively (without fermentation), to 5330.0 and 5690.0 mg·100 g^−1^, respectively (fermented with A. awamori), and 5140.0 and 5690.0 mg·100 g^−1^, respectively (fermented with A. oryzae). Furthermore, the authors reported that in all days of fermentation, a DPPH radical scavenging activity greater than 100% was observed, when compared to black rice bran without fermentation.

It is possible to explore the impact of solid-state fermentation on black rice bran using Aspergillus awamori and Aspergillus oryzae. They observed that fermentation led to increased levels of certain phenolic acids, such as protocatechuic acid and vanillic acid, as well as enhanced antioxidant activity. These findings suggest that solid-state fermentation could be a valuable method for enhancing the nutritional and functional properties of black rice bran. Future research could focus on optimizing fermentation conditions to further enhance the phenolic acid content and antioxidant activity of black rice bran, as well as exploring its potential applications in food and nutraceutical industries.

## 5. Health Benefits of Black Rice-Based Foods

Food products developed with black rice can show bioactivity with health-promoting effects, which can improve the nutritional value of food products due to the presence of bioactive molecules. The functionality of bioactive molecules in black rice food products is related to reducing the risk of several chronic diseases, since they can perform antioxidant, antihypertensive, antidiabetic, anticancer, anti-obesity, and cholesterol-lowering activities [5].

In this context, Aalim et al. [16] investigated the effect of heat treatments and subsequent cooking on the phenolic properties and total antioxidant activity of black rice. They found that raw grains had considerably higher levels of phenolic compounds compared to cooked grains. Thermal decomposition of anthocyanins and cyanidin-3-glucoside was evident throughout processing, and was reflected in increased levels of protocatechuic acid, while proanthocyanidins were susceptible to cooking. The heat-treated grains exhibited substantial activity against α-amylase (8.36 mg mL^−1^), α-glucosidase (19.37 mg mL^−1^), and glycation (4.48 mg mL^−1^), while their cooked counterparts reduced the estimated glycemic index and increased resistance. These results emphasize the beneficial effect of black rice processing on nutritional management in diabetes and hyperlipidemia [13].

Additionally, Shozib et al. [26] conducted the antioxidant characterization of black rice cultivars in baked products. The content of cyanidin-3-glucoside was reduced by 48.05% in cooked rice compared to raw rice. Formulating baked food products using black rice flour also retained a higher content of cyanidin-3-glucoside (19.6 mg·100 g^−1^) compared to cooked rice products. This study demonstrated that black rice flour can be used as an ingredient in baking products, where it can retain more cyanidin-3-glucoside than by simply cooking.

On the other hand, Lang et al. [23] evaluated the influence of adding transglutaminase on the content of phenolic compounds in black rice cake. Transglutaminase enzyme is applied in gluten-free products to simulate the gluten network in terms of gas retention capacity. The total content of phenolic compounds in black rice cakes decreased by 9.5% after cooking. Individually, ferulic and p-coumaric acids were the only phenolic acids whose amounts decreased after cooking, with variations of 43.5 and 34.9%, while other phenolic acids and flavonoids, such as quercetin and catechin, increased in extractability after cooking, with variations of 3.3 and 0.0%. Thus, they observed that black rice cakes had the highest total phenolic compound content, and only had a reduction in ferulic and p-coumaric acid content compared to other rice varieties.

Mau et al. [27] produced chiffon cakes with substitution of 0 to 100% (*w*/*w*) of wheat flour with black rice flour. Chiffon cake is a popular ready-to-eat food enjoyed by consumers worldwide, and would be a good way to ingest bioactive compounds. The chiffon cake formulated with partial substitution of wheat flour by up to 60% black rice flour contained more bioactive components (66.968 mg GAE·100 g^−1^) than the control cake (34.124 mg GAE·100 g^−1^). Moreover, the black rice chiffon cake showed good antioxidant activity (6.114 mg extract.mL^−1^).

Papillo et al. [28] extracted polyphenols rich in anthocyanins from black rice to produce functional foods, such as cookies. The cookies enriched with anthocyanins showed significantly higher levels of polyphenols, antioxidant capacity, and anthocyanins compared to the control cookies. The polyphenolic extract from black rice can be regarded as a valuable source of polyphenols for producing functional foods or microencapsulated ingredients for nutraceutical applications.

Food products incorporating black rice offer various health benefits due to their bioactivity, which includes antioxidant, antihypertensive, antidiabetic, anticancer, anti-obesity, and cholesterol-lowering effects. Studies have shown that heat treatments and cooking affect the phenolic properties and antioxidant activity of black rice, with raw grains containing higher phenolic compounds than cooked ones. Baked products with black rice flour retain more cyanidin-3-glucoside than cooked rice alone, suggesting its potential as a baking ingredient. Additionally, black rice cakes have been found to have the highest total phenolic compound content, with certain phenolic acids decreasing after cooking while others increase. Chiffon cakes with black rice flour substitution contain more bioactive components than control cakes, with good antioxidant activity. Extracted polyphenols from black rice, particularly anthocyanins, show promise for use in functional foods or nutraceutical applications. Further research could explore the development of more black-rice-based functional foods and their impacts on human health.

## 6. Conclusions and Final Considerations

The studies on black rice have revealed valuable insights into its phytochemical distribution and nutritional significance. Outer layers of black rice grains, particularly the rice bran, contain higher concentrations of phenolic compounds, flavonoids, and anthocyanins compared to inner layers, highlighting their nutritional importance. The comparison with brown rice further underscores the nutritional superiority of black rice, which exhibits potential health benefits such as antioxidant, antihypertensive, antidiabetic, anticancer, anti-obesity, and cholesterol-lowering activities. Not only post-harvest processes, such as drying and storage, but also different processing methods, such as cooking, roasting, and baking, impact the bioactive compounds differently, with some compounds decreasing and others increasing. However, the overall impact of processing on the bioactive molecules in black rice suggests its potential as a functional food with industrial applications. Future research could focus on optimizing processing techniques to preserve or enhance the nutritional profile of black rice products and explore their health effects in human trials. Developing innovative processing methods could lead to the production of healthier and more nutritious food products.

## Figures and Tables

**Table 1:** Total phenolics, total flavonoids, total anthocyanins, antioxidant activity, and individual free phenolics of processed black rice.

Processing	Condition	Total Phenolics	Total Flavonoids	Total Anthocyanins	Antioxidant Activity	Individual Free Phenolics	References
Drying	20–100 °C in fixed bed dryer	807.0–880.0 mg·100 g^−1^	290.0–328.0 mg·100 g^−1^	400.0–450.0 mg·100 g^−1^	-	Caffec acid: 10.2–14.9 mg·100 g^−1^ C3G: 12.0–39.0 mg·100 g^−1^ Ferulic acid: 12.0–31.0 mg·100 g^−1^ Gallic acid: 7.0–12.0 mg·100 g^−1^ p-coumaric acid: 14.0–19.5 mg·100 g^−1^ P3G: 14.4–16.0 mg·100 g^−1^ PAA: 26.0–37.0 mg·100 g^−1^ Quercetin: 31.0–35.0 mg·100 g^−1^	Lang et al. [6]
Drying	In a drum dryer	5974.0 mg GAE·100 g^−1^	1030.0 mg GAE·100 g^−1^	121.79 mg C3GE·100 g^−1^	30 mM TE·100 g^−1^ (ABTS assay)	C3,5G: 12.1 mg100 g^−1^ C3G: 177.0 mg·100 g^−1^ P3G: 21.2 mg·100 g^−1^ PAA: 26.4 mg·100 g^−1^ Syringic acid: 32.4 mg·100 g^−1^ Vanillic acid: 15.3 m. 100 g^−1^	Qiu et al. [13]
Storage	Normal, nitrogen, and vacuum atmospheres for 12 months	680.0–830.0 mg·100 g^−1^	280.0–445.0 mg·100 g^−1^	295.0–330.0 mg·100 g^−1^	-	Caffec acid:10.2–18.0 m. 100 g^−1^ C3G: 8.0–9.0 mg·100 g^−1^ Ferulic acid: 4.0–6.0 mg·100 g^−1^ p-coumaric acid: 7.0–8.5 mg·100 g^−1^ P3G: 10.8–12.4 mg·100 g^−1^ PAA: 21.5–34.0 mg·100 g^−1^ Quercetin: 8.0–16.0 mg·100 g^−1^	Lang et al. [6]
Storage	16–40 °C for 6 month	2210.0–2300.0 mg GAE·100 g^−1^	-	-	-	C3G: 21,837.0–21,868.0 mg·100 g^−1^ Ferulic acid: 16.0–63.0 mg·100 g^−1^ p-coumaric acid: 0.0–10.0 mg·100 g^−1^ Quercetin: 26.0–45.0 mg·100 g^−1^	Ziegler et al. [14]
Storage	UV-C exposition for 3 h and storage for 6 months	-	-	-	-	p-coumaric acid: 80.8–11.0 mg·100 g^−1^ Ferulic acid: 28.0–34.0 mg·100 g^−1^ Gallic acid: 10.0–11.0 mg·100 g^−1^ HBA: 25.0–53.0 mg·100 g^−1^	Ferreira et al. [15]
Cooking	180 °C for 20 min	475.0 mg·100 g^−1^	200.0 mg·100 g^−1^	260.0 mg·100 g^−1^	-	Gallic acid: 324.0 mg·100 g^−1^ PAA: 35,988.0 mg·100 g^−1^ Vanillic acid: 2229.0 mg·100 g^−1^ C3G: 11,618.0 mg·100 g^−1^ Ferulic acid: 565.0 mg·100 g^−1^ Vanillic acid: 2229.0 mg·100 g^−1^	Aalim et al. [16]
Roasting	180 °C for 20 min	575.0 mg·100 g^−1^	345.0 mg·100 g^−1^	160.0 mg·100 g^−1^	-	Gallic acid: 43.0 mg100 g^−1^ PAA: 2478.50 mg ·100 g^−1^ Vanillic acid: 2775.0 mg·100 g^−1^ C3G: 13,515.0 mg·100 g^−1^ Ferulic acid: 304.0 mg·100 g^−1^ Vanillic acid: 2775.0 mg·100 g^−1^	Aalim et al. [16]
Frying	140 °C for 5 min	430.0 mg·100 g^−1^	180.0 mg·100 g^−1^	50.0 mg·100 g^−1^	-	Gallic acid: 260.0 mg·100 g^−1^ PAA: 15,608.0 mg·100 g^−1^ Vanillic acid: 1867.0.mg 100 g^−1^ C3G: 2409.0 mg 100 g^−1^	Aalim et al. [16]
						Ferulic acid: 200.0 mg·100 g^−1^ Vanillic acid: 1867.0 mg·100 g^−1^	
Cooking	100 °C	624.4–1384.8 mg·100 g^−1^	-	-	-	C3G: 6310.0–10,060.0 mg·100 g^−1^ P3G: 330.0–670.0 mg·100 g^−1^	Melini et al. [10]
Boiling	18–20 min	-	-	0.8–3.9 mg·100 g^−1^	0.08–0.16 mM AAE·100 g^−1^ (ABTS assay)	Ferulic acid: 0.0–248.8 mg·100 g^−1^ Gallic acid: 454.5–688.0 mg·100 g^−1^ Kaempferol: 50.7–99.3 mg·100 g^−1^ Myricetin: 30.0–100.8 mg·100 g^−1^ PAA: 0.0–118.8 mg·100 g^−1^ Vanillic acid: 44.5–81.5 mg·100 g^−1^	Bagchi et al. [17]
Beating	60 °C for 45 min	-	-	16.72–33.09 mg·100 g^−1^	0.27–0.32 mM AAE·100 g^−1^ (ABTS assay)	Ferulic acid: 1224.0–1789.2 mg·100 g^−1^ Kaempferol: 56.3–192.4 mg·100 g^−1^ Myricetin: 116.2–310.0 mg·100 g^−1^ Gallic acid: 1099.1–1560.5 mg·100 g^−1^ PAA: 0.0–855.0 mg·100 g^−1^ Vanillic acid: 12.4–379.1 mg·100 g^−1^	Bagchi et al. [17]
Popping	>177 °C for 40–50 s	-	-	4.30–31.93 mg·100 g^−1^	0.29–0.32 mM AAE·100 g^−1^ (ABTS assay)	Gallic acid: 1592.5–2782.3 mg·100 g^−1^ Ferulic acid: 157.4–1635.5 mg·100 g^−1^ Kaempferol: 29.3–109.5 mg·100 g^−1^ PAA: 0.0–984.8 mg·100 g^−1^ Vanillic acid: 0.0–314.8 mg·100 g^−1^ Myricetin: 91.3–799.7 mg·100 g^−1^	Bagchi et al. [17]
Puffing	Roasted with 10–12% brine solution for 1–2 min at 220 °C	-	-	0.44–17.62 mg·100 g^−1^	0.14–0.26 mM AAE·100 g^−1^ (ABTS assay)	Gallic acid: 1009.2–1517.3 mg·100 g^−1^ Vanillic acid: 19.2–319.7 mg·100 g^−1^ Ferulic acid: 383.7–1272.8 mg·100 g^−1^ Kaempferol: 39.6–265.6 mg·100 g^−1^ PAA: 0.0–965.7 mg·100 g^−1^ Myricetin: 60.9–435.9 mg·100 g^−1^	Bagchi et al. [17]
Polishing	Using Satake rice huller and miller machine	-	-	3.50–57.23 mg·100 g^−1^	0.32–0.32 mM AAE·100 g^−1^ (ABTS assay)	Ferulic acid: 918.9–2933.0 mg·100 g^−1^ Gallic acid: 844.9–1871.8 mg·100 g^−1^ Kaempferol: 15.7–65.7 mg·100 g^−1^ Myricetin: 308.3–1688.6 mg·100 g^−1^ PAA: 0.0–751.1 mg·100 g^−1^ Vanillic acid: 0.0–242.7 mg·100 g^−1^	Bagchi et al. [17]
Extrusion	150 °C	180.71 mg FAE·100 g^−1^	153.71 mg CE·100 g^−1^	105.68 mg C3GE·100 g^−1^	0.87 mM TE·100 g^−1^ (DPPH assay)	-	Meza et al. [9]
Extrusion	-	11,375.0 mg GAE·100 g^−1^	6827.0 mg GAE·100 g^−1^	33.68 mg C3GE·100 g^−1^	144.0 mM TE·100 g^−1^ (ABTS assay)	C3,5G: 13.9 mg·100 g^−1^ C3G: 74.5 mg·100 g^−1^ P3G: 13.2 mg·100 g^−1^ PAA: 95.0 mg·100 g^−1^ Syringic acid: 38.9 mg·100 g^−1^ Vanillic acid: 18.5 mg·100 g^−1^	Qiu et al. [13]
Extrusion	40, 60, 80 e 120 °C	-	-	0.077 mg C3GE·100 g^−1^	2.2 mM TE·100 g^−1^ (ABTS assay)	Caffeic acid: 15.0 mg·100 g^−1^ Ferulic acid: 199.0 mg·100 g^−1^ HBA: 56.0 mg·100 g^−1^ p-coumaric acid: 129.0 mg·100 g^−1^ PAA: 14.0 mg·100 g^−1^ Sinapic acid: 156.0 mg·100 g^−1^ Syringic acid: 120.0 mg·100 g^−1^ Vanillic acid: 238.0 mg·100 g^−1^	Blandino et al. [15]
Steaming	Steamed for 15 min	26.0–49.0 mg·100 g^−1^	9.0–17.0 mg·100 g^−1^	-	10.0–17.0 mg PA·100 g^−1^ (DPPH assay)	-	Susmitha et al. [18]
Milling	30–75 s	168,139.0–443,612.0 mg GAE·100 g^−1^	430,139.0–1,310,616.0 mg GAE·100 g^−1^	789.50–3158.45 mg C3GE·100 g^−1^	38.8–145.8 mM TE·100 g^−1^ (ORAC assay)	C3G: 74,409.0–256,863.0 mg·100 g^−1^ Ferulic acid: 969.7–344.1 mg·100 g^−1^ Gallic acid: 92.3- 649.3 mg·100 g^−1^ p-coumaric acid: 63.4–502.7 mg·100 g^−1^ P3G: 187,100.0–95,460.0 mg·100 g^−1^ Quercetin: 139.6–599.7 mg·100 g^−1^ Vanillin: 81.2–124.2 mg·100 g^−1^	Zhang et al. [18]
Baking	With and without enzyme	49.1–55.1 mg·100 g^−1^	-	-	-	Caffeic acid: 26.0–29.0 mg·100 g^−1^ Ferulic acid: 1521.0–2060.0 mg·100 g^−1^ HBA: 141.0–152.0 mg 100 g^−1^ p-coumaric acid: 160.0–194.0 mg·100 g^−1^ PAA: 2933.0–2965.0 mg·100 g^−1^ Quercetin: 77.0–79.0 mg·100 g^−1^	Lang et al. [6]
Sprouting	25 °C and 95% of moisture for 3 days	77.0–149.0 mg·100 g^−1^	36.0–45.0 mg·100 g^−1^	890.7–4582.5 mg·100 g^−1^	0.06–0.045 mM TE·100 g^1^ (DPPH assay)	-	Islam et al. [19]
Fermentation	With *Aspergillus awamori* for 1–5 days	1200.0–2000.0 mg GAE·100 g^−1^	-	-	2.01–2.25 mM TE·100 g^−1^ (DPPH assay)	Caffeic acid: 28.0–183.0 mg·100 g^−1^ C3G: 49.9–103.7 mg·100 g^−1^ Ferulic acid: 382.0–5665.0 mg·100 g^−1^ HBA: 179.0–532.0 mg·100 g^−1^ p-coumaric acid: 185.0–569.0 mg·100 g^−1^ PAA: 4986.0–16,606.0 mg·100 g^−1^ Vanillic acid: 1884.0–4620.0 mg·100 g^−1^	Shin et al. [20]
Fermentation	With *Aspergillus oryzae* for 1–5 days	1200.0–2800.0 mg GAE·100 g^−1^	-	-	2.1–2.2 mM TE·100 g^−1^ (DPPH assay)	Caffeic acid: 38.0–122.0 mg·100 g^−1^ C3G: 66.6–108.9 mg·100 g^−1^ Ferulic acid: 440.0–4128.0 mg·100 g^−1^ HBA: 150.0–514.0 mg·100 g^−1^ p-coumaric acid: 149.0–449.0 mg·100 g^−1^ PAA: 4771.0–15,796.0 mg·100 g^−1^ Vanillic acid: 745.0–4453.0 mg·100 g^−1^	Shin et al. [20]

C3,5G, cyanidin 3,5-diglucoside; C3G, cyanidin-3-glucoside; P3G, peonidin 3-O-glucoside; PAA, protocatechuic acid; HBA, hydroxybenzoic acid; GAE, gallic acid equivalent; DPPH, 2,2-diphenyl-1-picrylhydrazyl; ORAC, oxygen radical absorbance capacity; ABTS, 2,2′-azino-bis-(3-ethylbenzothiazoline-6-sulfonic) acid; CE, catechin equivalent; TE, trolox equivalent; AAE, ascorbic acid equivalent; PA, phenolic acid (mixture of gallic acid, protocatechuic acid, p-hydroxybenzoic acid, vanillic acid, p-coumaric acid, caffeic acid, ferulic acid, and chlorogenic acid).

## Data Availability

Not applicable.

## Abbreviations

GAE: gallic acid equivalent; QE, quercetin equivalent; DPPH, 2,2-diphenyl-1-picrylhydrazyl; ORAC, oxygen radical absorbance capacity; CE, catechin equivalent; TE, trolox equivalent; PA, phenolic acid (mixture of gallic acid, protocatechuic acid, p-hydroxybenzoic acid, vanillic acid, p-coumaric acid, caffeic acid, ferulic acid, and chlorogenic acid); RE, rutin equivalent; FRAP, ferric reducing antioxidant power; ABTS, 2,2′-azino-bis-(3-ethylbenzothiazoline-6-sulfonic) acid.
